# Subject-Specific Computational Fluid-Structure Interaction Modeling of Rabbit Vocal Fold Vibration

**DOI:** 10.3390/fluids7030097

**Published:** 2022-03-06

**Authors:** Amit Avhad, Zheng Li, Azure Wilson, Lea Sayce, Siyuan Chang, Bernard Rousseau, Haoxiang Luo

**Affiliations:** 1 Department of Mechanical Engineering, Vanderbilt University, 2301 Vanderbilt Place, Nashville, TN 37235, USA; 2 Department of Communication Science and Disorders, University of Pittsburgh, 4200 Fifth Avenue, Pittsburgh, PA 15260, USA

**Keywords:** fluid-structure interaction, subject-specific model, vocal fold vibration, 3D model, phonation, biomechanics

## Abstract

A full three-dimensional (3D) fluid-structure interaction (FSI) study of subject-specific vocal fold vibration is carried out based on the previously reconstructed vocal fold models of rabbit larynges. Our primary focuses are the vibration characteristics of the vocal fold, the unsteady 3D flow field, and comparison with a recently developed 1D glottal flow model that incorporates machine learning. The 3D FSI model applies strong coupling between the finite-element model for the vocal fold tissue and the incompressible Navier-Stokes equation for the flow. Five different samples of the rabbit larynx, reconstructed from the magnetic resonance imaging (MRI) scans after the in vivo phonation experiments, are used in the FSI simulation. These samples have distinct geometries and a different inlet pressure measured in the experiment. Furthermore, the material properties of the vocal fold tissue were determined previously for each individual sample. The results demonstrate that the vibration and the intraglottal pressure from the 3D flow simulation agree well with those from the 1D flow model based simulation. Further 3D analyses show that the inferior and supraglottal geometries play significant roles in the FSI process. Similarity of the flow pattern with the human vocal fold is discussed. This study supports the effective usage of rabbit larynges to understand human phonation and will help guide our future computational studies that address vocal fold disorders.

## 1. Introduction

The voice production process, or phonation, involves flow-induced vocal fold vibration and is a complex nonlinear interplay between the glottal aerodynamics and the structural dynamics of the elastic vocal fold tissue. The study of this fluid-structure interaction (FSI) problem will be useful in the understanding of its complex biological process as well as in the applications of surgical plannings, diagnosis, and tool development [1-5].

The practical challenges in the direct study of the human larynx demands to opt for alternate methods like the experimentation on other mammals and computational modeling. There were many numerical studies previously carried out on this topic, which have contributed largely to the understanding of the basic biomechanics behind phonation, but there is still a shortage of integrated experimental/computational studies for the in vivo phonation that advance the computational modeling techniques toward practical clinical applications. For brevity, only the most recent efforts on computational phonation modeling are discussed here to provide the context for the current study.

Most of the earlier computational models focused on idealized and generic representations for the laryngeal geometry. These models include structural models utilizing the finite element method and coupled with a one-, two-, or three-dimensional (1D, 2D, or 3D) flow solver to simulate the FSI process [6-13]. Many lessons have been learned from these insightful studies, including the vibratory characteristics of the vocal fold tissue, the pulsatile jet flow behavior, the transfer of momentum and energy from the flow to the solid, and the multi-faceted effects of the geometric and material properties. Acoustic wave propagation and interaction with the vocal fold were sometimes included in the computational model as well [5,14,15]. In addition to computational studies, there have also been many experimental studies of the FSI process of vocal fold using mechanical models or excised larynges [16-22]. Readers are encouraged to refer to Mittal et al. [3] to get an overview on the status of the computational phonation modeling and the need for more research efforts pertaining to the topic.

The gross simplifications of the laryngeal models in the previous studies lacks specificity in the phonation dynamics for individual subjects and thus have limited applications for the subject- or patient-specific treatment of voice disorders. In order to lead the current computational technology toward those special applications, more efforts in incorporating the important subject-specific information in the computational model and substantial integrated experimental/computational studies to test and validate the computational models are required.

The advancement of medical imaging technologies such as magnetic resonance imaging (MRI) have permitted the subject-specific features in the laryngeal geometry. For example, Wu and Zhang [23] used an MRI derived human vocal fold model to perform a study of the geometric parameters. A recent study by Xue et al. [24] presented a 3D FSI simulation using the subject-specific geometry of the human larynx based on MRI data. This research particularly highlighted the importance of the use of actual geometries as compared with simplistic models in the previous FSI studies. The authors pointed out the asymmetry observed in the flow in the supraglottal region when viewed in the mid-sagittal plane. This flow behavior was mainly attributed to the asymmetry in the geometry. A similar study was carried out for avian phonation using subject-specific geometries [14]. Despite their significant advancement, these studies have the limitation of not being able to prescribe the individual characteristics of the vocal fold tissue’s material properties, and the tissue specifications were primarily based on the general population’s data. In addition, generally there has been a lack of available experimental validation for subject-specific computational models of vocal fold in the literature.

One of the reasons for very few studies on the subject-specific FSI study of the vocal fold is the high computational cost to simulate the problem. Even with the availability of high-performance computers for fast calculations, the complex anatomy of the larynx and vocal fold movement pose a huge challenge for meshing in the 3D computational fluid dynamics of the glottal airflow. Another major challenge is the uncertain material properties, which could be significantly different among individuals. Within a species, every individual vocal fold has unique material properties [25,26]. This unavailability of the individual tissue properties restricts the validity of a subject-specific FSI model. In addition to these difficulties on the modeling side, setting up corresponding experiments to test and validate the subject-specific computational model has its own challenges, especially for in vivo phonation, e.g., obtaining the 3D scan data and high-speed images of vocal fold vibration in addition to managing the vocal fold adduction.

In our recent work, we have demonstrated the ability to integrate the in vivo phonation experiment using rabbits and perform corresponding subject-specific FSI modeling of the vocal fold vibration [27]. In that study, live rabbits were used for evoked phonation tests with simultaneous high-speed imaging and subglottal pressure measurement [28]. The larynx was excised after the tests for MRI scan and geometric reconstruction. The finite-element model (FEM) of the vocal fold tissue was then built for FSI analysis. Furthermore, the tissue stiffness properties were adjusted in a simplified FSI model, i.e., 1D flow coupled with 3D FEM, to match the vibration frequency of each individual sample. The resulting FSI model therefore has both geometric and material features of individual subjects. The current study is built upon our previous work [27] to perform further 3D simulation analyses of such subject-specific model and investigate the flow and vocal fold behavior. This investigation will provide in-depth understanding of rabbit phonation and therefore lead to constructive discussion of using rabbits as an alternative to human study. In addition, we will also compare the 3D simulation results with the results based on a recently developed, machine-learning enhanced 1D glottal flow model to assess the performance of the simplified flow model.

## 2. Modeling Methods and Case Setup

### 2.1. Summary of the Previous Phonation Experiment

**Figure 1.**
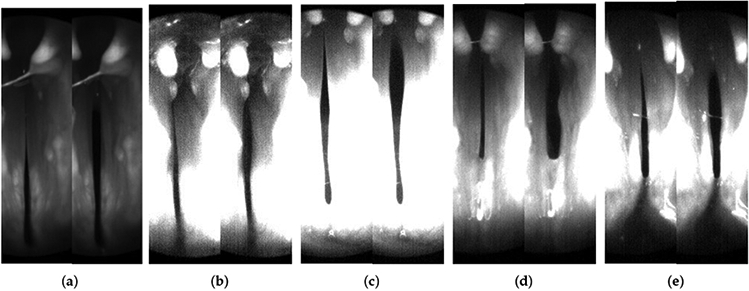
Images of the glottis during phonation (supraglottal view) from the high-speed camera for the five samples 1 to 5 in (**a–e**) respectively. The left image for every sample represents the vocal fold closure, and the right side represents the vocal fold opening.

The experiments were conducted on five male New Zealand white breeder rabbits under the approval of Vanderbilt University Institutional Animal Care and Use Committee. Readers can refer to Novaleski et al. [28], Ge et al. [29], and Swanson et al. [30] for detailed experimentation procedures and objectives. A bilateral Isshiki-type IV thyroplasty was performed, involving suturing of thyroid and cricoid cartilages to bring together the two sides of the vocal fold. This procedure enables in vivo rabbit phonation when a continuous humidified airflow is delivered through the glottis. A high-speed camera recorded the supraglottal view of vocal fold vibration at 10,000 frames-per-second (FPS). During this process, subglottal pressure and flow rate were also recorded. Figure 1 shows images of the glottis from the high-speed camera for each larynx sample.

### 2.2. Subject-Specific Models of the Vocal Fold

**Figure 2.**
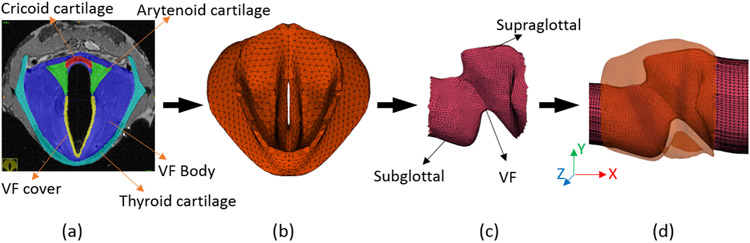
(**a**) Manual segmentation of the MRI data for the vocal fold (VF). (**b**) Reconstructed 3D VF mesh model. (**c**) Extracted lumen from the 3D larynx. (**d**) Extensions added in both directions as the flow domain (only a small portion of inlet and outlet are shown).

The phonation experiment was followed by larynx excision and the MRI scan. The scan provided morphology details of the vocal fold at the adducted position. Through manual segmentation of the MRI images, the 3D geometrical model of the subject-specific larynx was constructed. The software ITK-SNAP was used for the segmentation. The solid model includes important anatomical features such as the vocal fold and its lamina propria layer, the ventricular fold, part of the trachea, and part of the supraglottal region. After smoothing the manually segmented geometry using the Gaussian image tool, a tetrahedral body mesh was achieved using ANSYS ICEM. The thin cover of the vocal fold was determined roughly based on its thickness identified from the MRI. A mesh-refinement test with a static load and finer mesh near the vocal fold cover resulted in an error of only 1.58%. This mesh model was used as the finite-element model of the vocal fold tissue. The 3D model was then processed in MATLAB to extract the lumen as the flow domain, which was then extended in both subglottal and supraglottal directions to incorporate the inlet and exit flow through the larynx. The entire process is shown in Figure 2, where the vocal fold is seen to surround the flow domain except for the inlet and outlet extensions.

**Figure 3.**
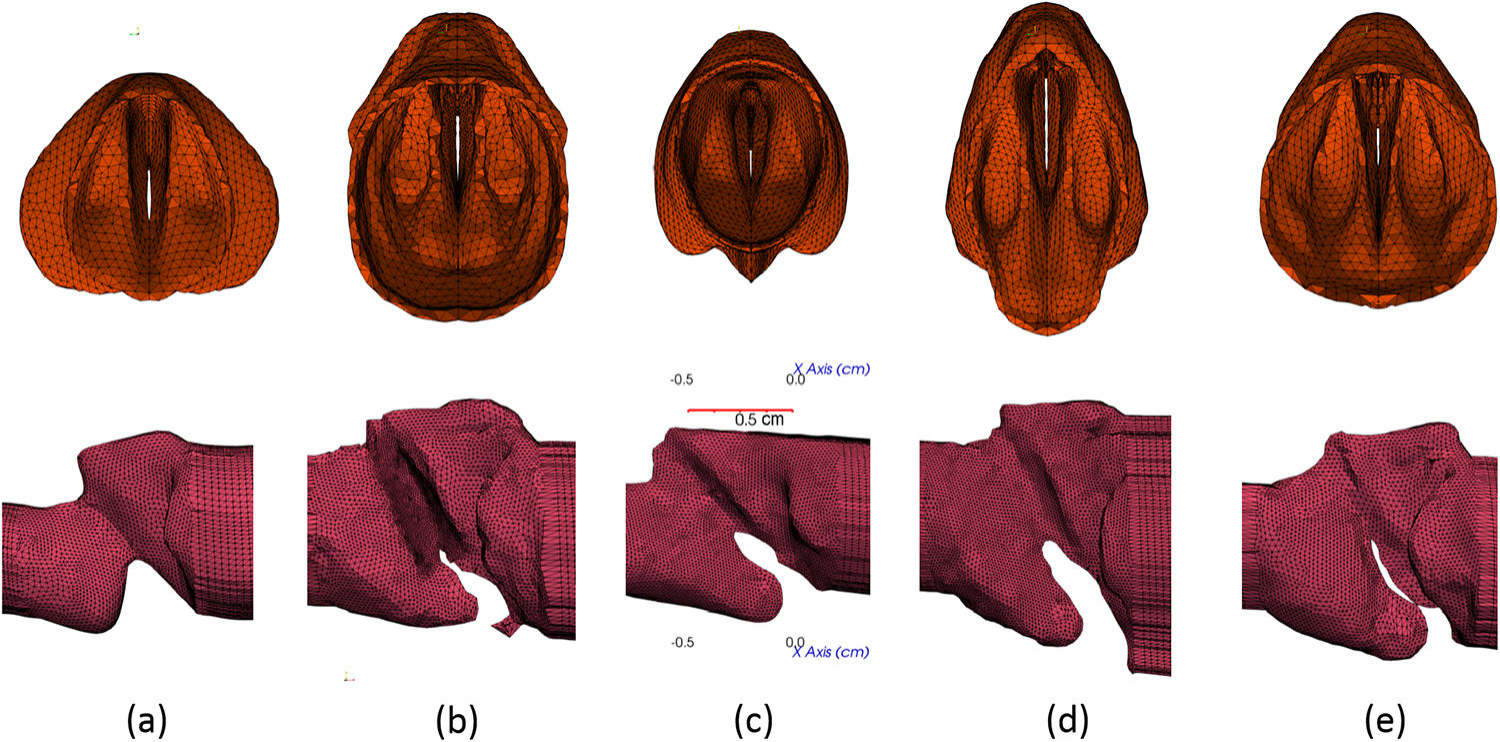
**Top row:** the reconstructed larynx models for all samples as viewed from the supraglottal side for samples 1 to 5 in (**a–e**) respectively; **bottom row:** the corresponding extracted lumen. A scale of 0.5 cm is shown in (**c**).

All five larynx samples have distinct geometries, which are shown in Figure 3 for comparison. Table 1 lists the total number of tetrahedral elements in each vocal fold. The finite-element model has all the exterior surfaces with the fixed boundary condition, whereas the lumen surface from the inside has a free boundary subject to the forces from the fluid. The vocal fold tissue is considered isotropic and described by the Saint Venant- Kirchoff model, which includes the finite strain for possible large displacements and rotation [31,32]. For each sample, Chang et al. [27] used an iterative method to determine the stiffness properties for the vocal fold body and lamina propria. That is, Young’s moduli were determined by matching the vibration frequency and amplitude between the 1D-flow based FSI simulation result and the phonation experiment. The tissue density and Poisson’s ratio are *ρ_s_* = 1000 kg/m^3^ and 0.3 respectively. The air density is at *ρ* = 1.0 kg/m^3^, and the air viscosity is increased by four times to reduce the Reynolds number and the demand for the simulation.

### 2.3. Governing Equations and Computational Methods for the FSI

The flow in the 3D FSI study is governed by the 3D viscous incompressible Navier–Stokes equation. We use a partitioned method to couple the flow solver and the finite-element solver of the tissue mechanics [31], where the two solvers are independent and retain their own modular form. At each time step, the two solvers are iterated until convergence is reached. For the flow solver, a Cartesian grid based immersed-boundary method is used to handle the complex moving boundaries, and this method has been validated extensively in our previous work [31,33].

In a recent work, we also developed a 1D pulsatile flow model for glottal airflow that incorporates machine learning [34]. In this model, the continuity and momentum equation are written as

(1) $$\frac{\partial A}{\partial t}+\frac{\partial Au}{\partial x}\;\;=\;0\;\rho \frac{\partial u}{\partial t}+\rho u\frac{\partial u}{\partial x}\;\;=\;-\frac{\partial p}{\partial x}+\frac{\partial \tau }{\partial x}$$

where *u*(*x*, *t*) and *p*(*x*, *t*) are the flow velocity and pressure, respectively, *A* is the effective cross-sectional area of the vocal fold lumen, and *τ* represents the pressure loss due to viscous and flow separation effects. Since we consider the entrance effect as the flow enters the narrow glottis from the subglottal region, *A* is based on correction of the actual cross-sectional area [35,36]. We used a machine learning strategy to determine the entrance effect and the pressure loss as non-dimensional functions of the Reynolds number and a few characteristic geometrical parameters of the instantaneous glottal shape. Further detail of this model can be found in our previous work [34] and is summarized in the Appendix A for convenience.

### 2.4. Simulation Setup

The flow domain in the 3D FSI is discretized using a non-uniform Cartesian grid, on which the mesh is stretched in the lateral (*Z* in Figure 2) direction so that it is finest in the glottis. For each sample, the mesh is approximately 200 in the axial direction, 130 in the lateral direction, and 130 in the anterior-posterior direction. The rectangular flow domain is decomposed into multiple subdomains to facilitate the parallel processing of the simulation. We have used approximately 65 to 133 processors for each sample, and the simulations were performed on Stampede2 at the Texas Advanced Computing Center (TACC). The time step used was Δ*t* = 10^−4^ centisecond to achieve good temporal resolution and ensure FSI stability, which leads to approximately 3000 time steps to resolve one vibration cycle. A mesh convergence study is provided in the Appendix B.

The subglottal pressure is set to be constant and the same as in the phonation experiment, and the exit pressure is set to be zero. Each sample has a different inlet pressure, as shown in Table 2. In addition, Young’s modulus of the vocal fold body *E_b_* and for the cover *E_c_* are given in Table 2. These parameters are used exactly as in the previous study on the 1D flow based FSI analysis by Chang et al. [27]. In both the 3D FSI and 1D-flow based FSI simulations, the flow is driven by the subglottal pressure, and the pulsatile flow is established automatically once the vocal fold vibration is induced in the process.

## 3. Results and Discussions

### 3.1. Vibration and Frequency Analysis

**Figure 4.**
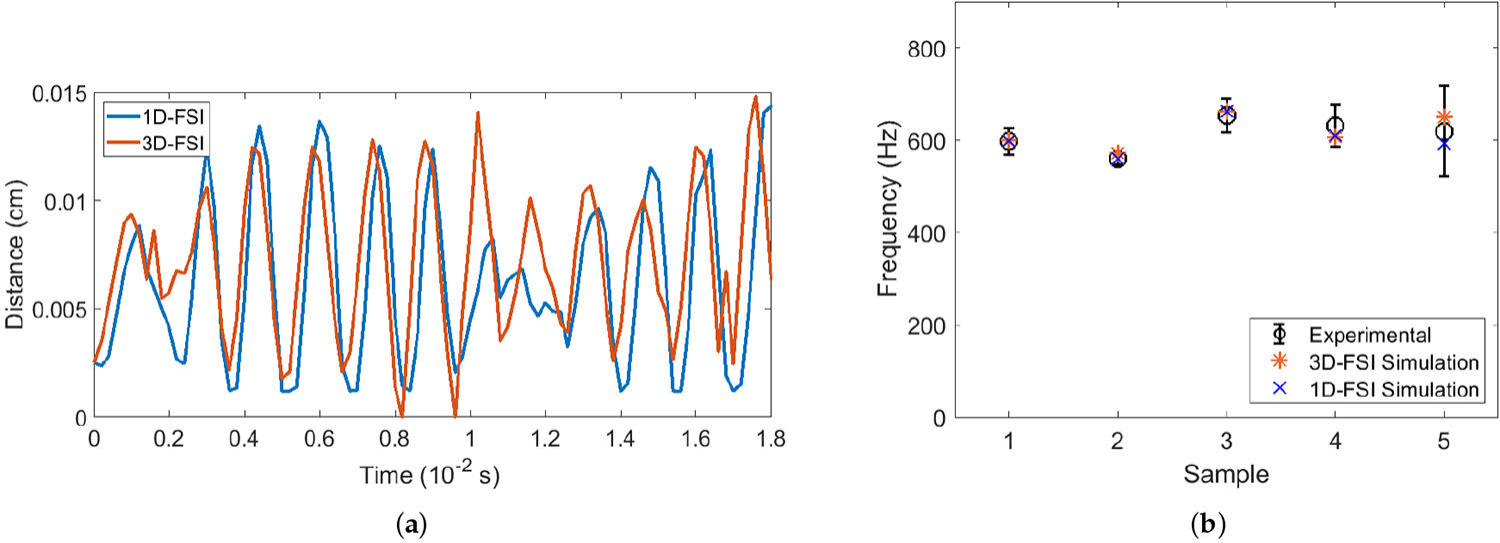
(**a**) VF vibration for Sample 3 plotted for the mid-glottis point in both 1D FSI model (blue line) and 3D FSI model (orange line). (**b**) VF vibration frequency comparison of all five samples among the experiments, 1D-FSI and 3D-FSI simulations.

The vocal fold vibrations were observed to be fairly symmetric between the left and right sides in the experiments. Thus, a marker point on one of the sides of the vocal fold around the halfway between the anterior and the posterior of the glottis is chosen from the output to reflect the vibration amplitude. Figure 4a shows the vibration for this point for Sample 3. For comparison, we have included both the 3D FSI simulation result and 1D-flow based FSI result from our previous work [34]. Within the time frame of 1 centisecond (cs), approximately five to six vibration cycles are clearly visible for the sample. The vibration waveform agrees well for the two FSI models.

Some differences in the vibration amplitude and the phase between the two FSI models can be seen and are understandable, since the accurate characteristics of vibration are dependent on the detail of the flow that is captured only in the 3D simulation. Indeed, it has been shown previously by Luo et al. [33] that the fluid forces on the vocal fold could be fluctuating due to the vortices in the flow. For all the samples, the vibrations are established when simulations are extended. We have calculated the vibration frequency exhibited by each sample using the Fast Fourier Transform (FFT) and compared the result with the previously published data from the experiments and 1D-flow FSI simulation, as shown in Figure 4b. We see a good agreement between the frequencies reported from the experiments, the 1D-flow FSI model, and the current 3D FSI model. Note that the vibration frequencies for all the five samples are in the range of generic vibration frequency for rabbits from experiments by other groups [22].

### 3.2. Power and Energy Analysis

In our numerical study, the flow is driven by a constant subglottal pressure at the inlet, which acts as the source of energy for the phonation process. In addition to providing the kinetic energy to the flow, part of the energy source is transferred to the vocal fold tissue through the stresses on the vocal fold surface and the tissue’s dynamic deformation. To calculate the power transfer or rate of work, *Ė*, from the flow to the tissue, we integrate the product of the fluid stress, **f**, which includes the shear and normal stresses, and the velocity of the vocal fold surface, **v**, so that *Ė* = ʃ **f** · **v** d*S*, where *S* is the vocal fold surface.

**Figure 5.**
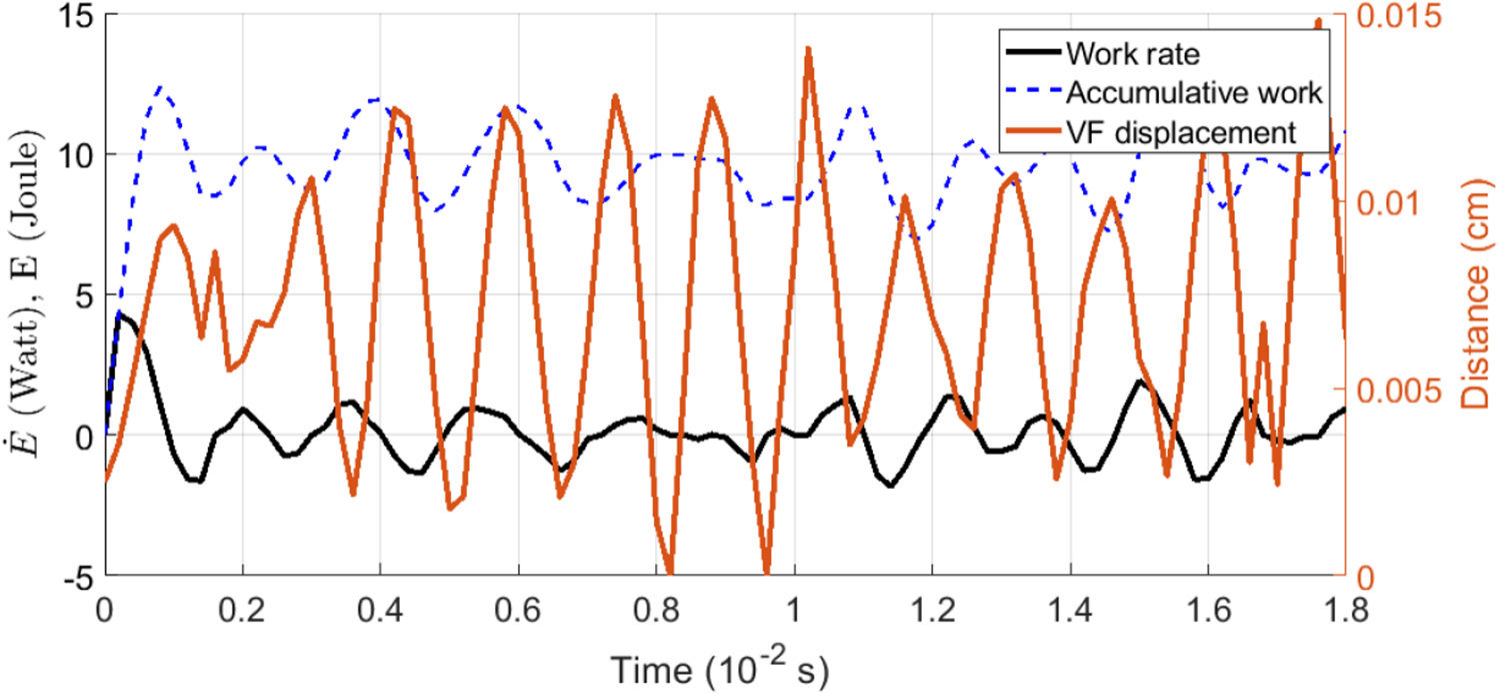
Rate of work, accumulative work, and VF vibration for Sample 3.

Figure 5 shows the work rate *Ė* along with the corresponding vocal fold vibration cycles for Sample 3. In the plot, we notice that the first opening phase (from stationary) consumes the maximum amount of power and then the instantaneous work rate oscillates between positive and negative values. The accumulated work *E* is also shown as a reference. From the figure, the first cycle shows a relatively large amount of work being transferred to the tissue, representing the initial opening of the vocal fold. Then, both *Ė* and *E* start to oscillate around their mean values, indicating the elastic recoil of the tissue structure. The average of *Ė* is small since the tissue is elastic and only a small amount of damping is added to the tissue. Further inspection shows that *Ė* is neither exactly in phase nor out of phase with the vibration.

**Figure 6.**
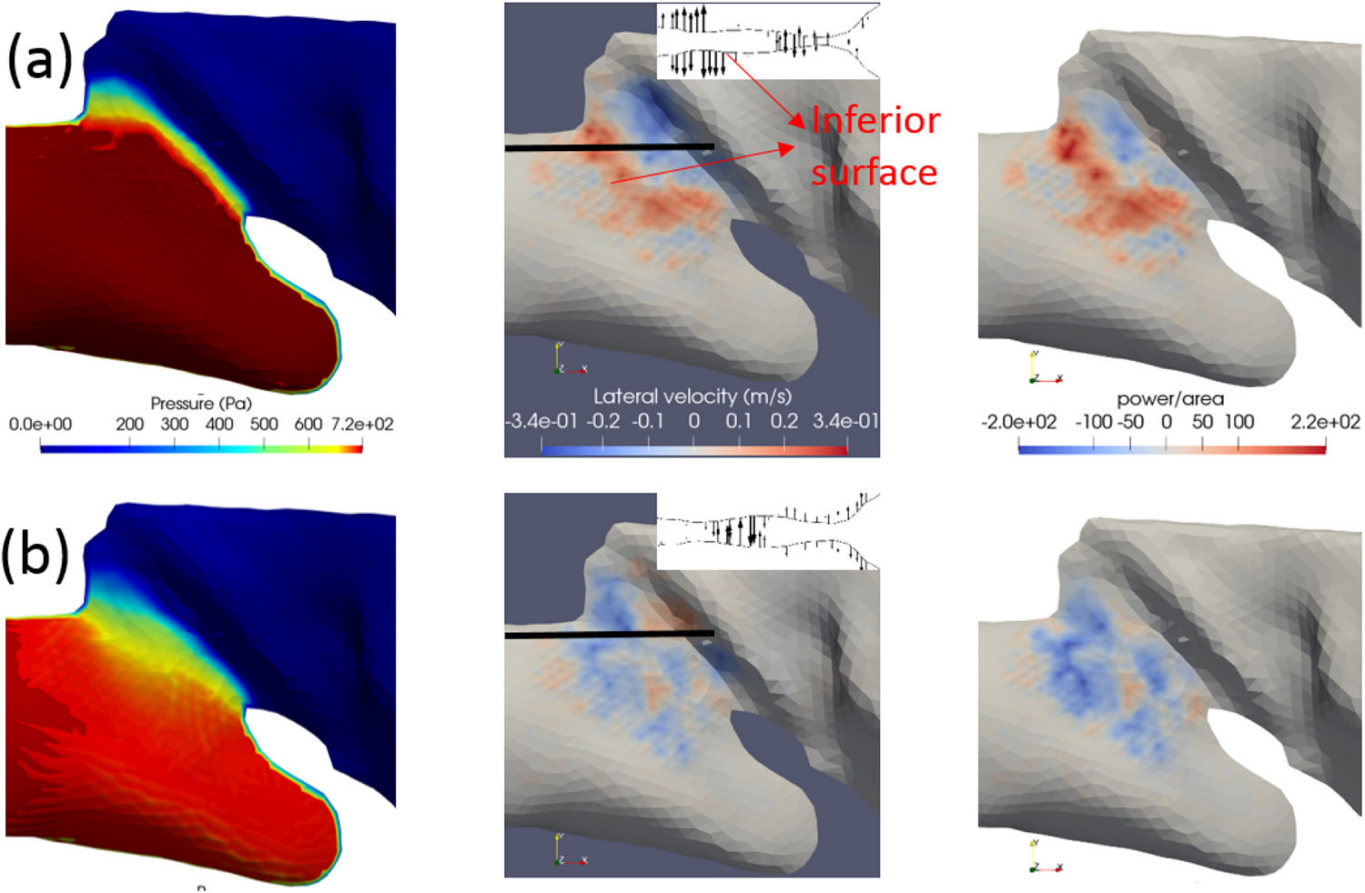
Contours on the vocal fold surface for Sample 3 at (**a**) *t* = 1.5 cs (VF closed) and (**b**) *t* = 1.6 cs (VF open). Left to right: pressure contour, lateral velocity *w*, power per unit area on the vocal fold surface. The inset on the center picture shows the vocal fold profile and the velocity vectors in the cut plane indicated by the thick bar in the picture.

To better understand the energy transfer and the vocal fold vibration, in Figure 6 we plot the instantaneous pressure and velocity contours along with the contours of the power per unit area, **f** · **v**, over the vocal fold surface at times *t* = 1.5 cs and *t* = 1.6 cs. As seen from Figure 5, *Ė* is positive at *t* = 1.5 cs when the vocal fold is closing and is negative at *t* = 1.6 when the vocal fold is near maximum opening. The power contours in Figure 6 show that most of the work is contributed by the sub-glottal surface for both time frames. Note from the inset in this figure that the vocal fold has a gentle slope and thus a large surface area subject to the pressure force. The corresponding pressure contours in Figure 6 show that the surface pressure is predominantly positive in the inferior region (prior to the glottal gap), and at the glottal gap the pressure level is rapidly reduced. This pressure distribution also facilitates most of the energy transfer through the inferior slope while this area of surface is deforming. Furthermore, since the pressure is positive at the inferior region, the sign of power contours is consistent with the sign of the lateral velocity of the vocal fold surface, *w*, as seen in Figure 6.

The insets in Figure 6 further show the instantaneous deformations of the vocal fold in an *XZ* cut plane at these two time moments. See Supplementary material for an animation of the vibration. At *t* = 1.5 cs, the glottis is closing but the inferior region is expanding quickly, leading to overall a positive rate of work; while at *t* = 1.6 cs, the inferior region is contracting quickly even though the glottis is nearly fully open, which leads to overall a negative rate of work. The velocity vectors in the insets indicate that the inferior region movement has a phase difference from the glottis opening/closing. As a result, the rate of work also has a corresponding phase difference from the glottal vibration, as seen in Figure 5. Similar inferior geometry and pattern of the surface power distribution are also observed for the other samples. Therefore, from these results we learn that the region has an important impact on the energy transfer of the rabbit vocal fold vibration. The effect of the inferior surface slope on the vocal fold vibration and energy transfer was discussed in a previous work using idealized geometries [37]. The present finding of the rabbit phonation regarding the inferior surface is consistent with the conclusion in that work.

**Figure 7.**
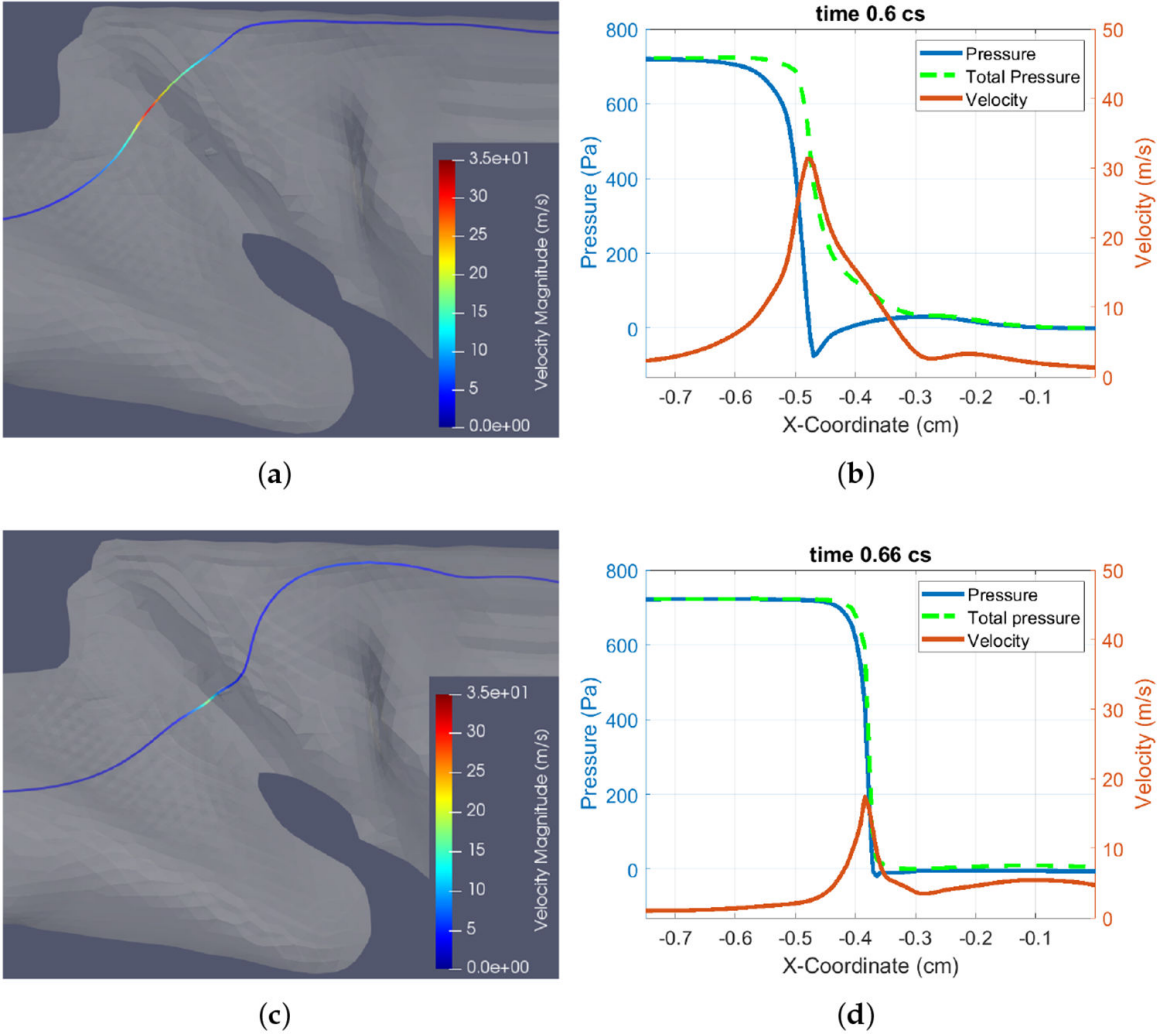
Change of the total pressure along a streamline at open or closed state for Sample 3. (**a**) A streamline at open state with t = 0.6 cs, and (**b**) the plots for *p*, *V*, and *p_total_* along this streamline; (**c**) A streamline at closed state with t = 0.66 cs, and (**d**) plots for *p*, *V*, and *p_total_* along this streamline.

In addition to the energy transfer from the fluid to the tissue, it is also important to examine the loss of total pressure along the flow, *p_total_* = *p* + 0.5*_ρ_V*^2^. Since the rabbit vocal fold, as seen in Figure 6, has a long, gradually converging inferior channel but a short and quickly diverging superior channel, it is expected that flow separation could be strong and the loss of total pressure is significant in the supraglottal area. To see this, from the 3D flow field we extract a streamline that goes through the glottis as seen in Figure 7a,c for Sample 3 at time *t* = 0.6 cs when the vocal fold is open and at *t* = 0.66 cs when the vocal fold is closed. The pressure and the velocity magnitude *V* along this streamline are plotted in Figure 7b,d.

It can be seen from Figure 7b, where the vocal fold is open, that the total pressure drops quickly from the glottis to the supraglottal region. Before the glottis, the total pressure is well preserved. The peak velocity magnitude on the streamline in this figure is 32 m/s around *x* = −0.48 cm, which is inside the glottal gap. At the same position the pressure drops to the minimum value, *p* = −75 Pa. According to the Bernoulli’s equation, the maximum flow speed should be at *V* ≈ 35 m/s, using the inlet pressure *P_in_* = 0.72 kPa and air density *ρ* = 1.0 kg/m^3^, which is close to the simulated value considering the viscous effect. In addition to the large expansion ratio from the thin glottal gap to the wide supraglottal region, another factor that could have affected the total pressure loss in the supraglottal region is that the overall shape of the lumen is curved and the flow impinges on the anterior wall in the supraglottal region. This feature may have caused additional losses in the flow.

When the vocal fold is closed, as in Figure 7d, the flow velocity is low, and the total pressure follows the pressure and quickly drops to nearly zero when reaching the glottis. This result is expected since the flow is nearly shut off and the supraglottal region is dominated by large flow circulation as seen later in the 3D visualization. The total pressure of the remnant flow is nearly all lost after exiting the glottis.

### 3.3. Pressure Comparison between 1D and 3D FSI

**Figure 8.**
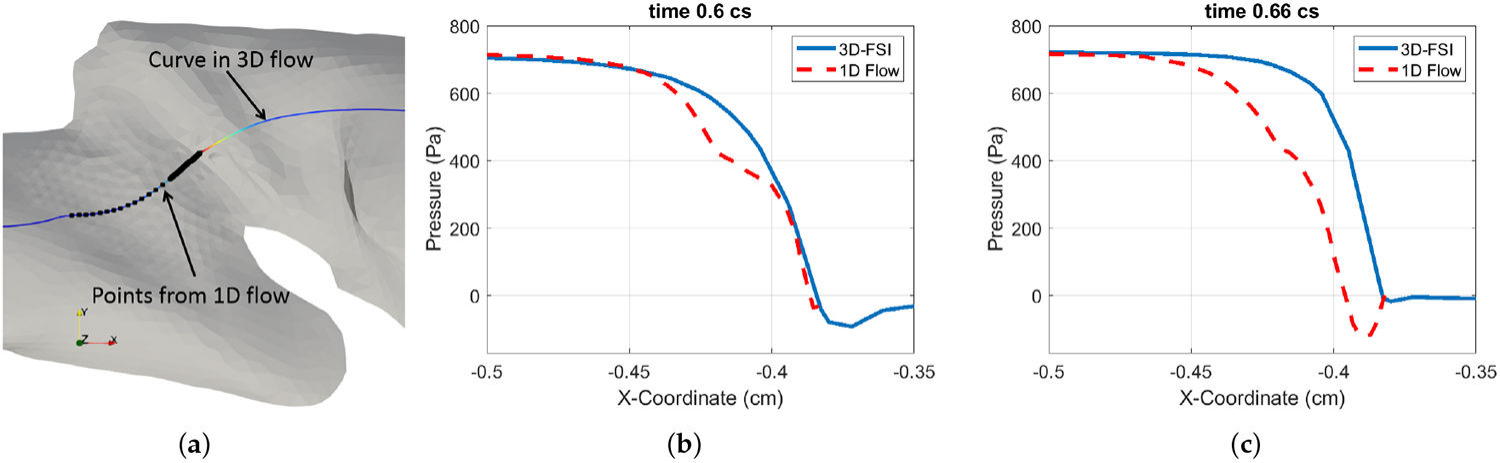
(**a**) Sample 3 model showing a streamline and the points (black markers) on it, along with the 1D flow model applied. (**b,c**) Pressure plot on the points from the 1D flow model (dashed) and 3D FSI model at an open state (*t* = 0.6 cs) (**b**) and closed state (*t* = 0.66 cs) (**c**).

The comparison of the vocal fold vibration in Section 3.1 shows that the 1D-flow based FSI produces consistent vibration pattern of the vocal fold as in the current 3D FSI simulation. To further examine the performance of this general 1D flow model, we compare the intraglottal pressure predicated by the 1D flow model with that extracted from the 3D flow field. To do the comparison, we first extract a streamline through the glottis, as shown in Figure 8a for an opening or closing state. The arc length of this streamline is used as the *x*-coordinate in the 1D flow model in Equation (1). The sequence of the dynamic vocal fold shapes from the 3D FSI simulation is used to calculate the cross-sectional area in the 1D flow model, which is subject to the correction due to the entrance effect [34]. The 1D flow model, which includes its machine learning derived functions for the pressure loss and entrance effect, is then used to calculate the pressure along the same streamline.

Figure 8b,c shows such comparison of the flow pressure between the 3D FSI simulation and the 1D flow model. In Figure 8b, the time is *t* = 0.6 cs, and the vocal fold is open; in Figure 8c, the time is *t* = 0.66 cs, and the vocal fold is closed. In both of these cases, it can be seen that the 1D flow based pressure calculation generally agrees with the pressure from the 3D simulation. Further inspection shows that at the open state, the 1D flow model slightly under-predicts the pressure prior to the narrowest gap; at the closed state, the difference is more pronounced, and the 1D flow shows lower pressure in the inferior region and some negative pressure in the glottal gap. Fortunately, such a difference did not significant impact the vocal fold vibration during the 1D flow based FSI simulation since the closed state is quite short in a vibration cycle.

### 3.4. Flow Field Analysis

One of the major objectives of this study is to visualize and analyze the 3D flow in the rabbit phonation. This was not done previously in Chang et al. [27], who focused mainly on the reduced-order modeling for the FSI. There has been limited research that employed subject-specific larynx models in the 3D FSI analysis. A few previous experimental studies [16,20,38] have found that in general the downstream flow in the supraglottal region is highly 3D. In the numerical study by Xue et al. [24], it was found that significant asymmetry in the flow exists in the supraglottal region for the subject-specific human larynx model.

**Figure 9.**
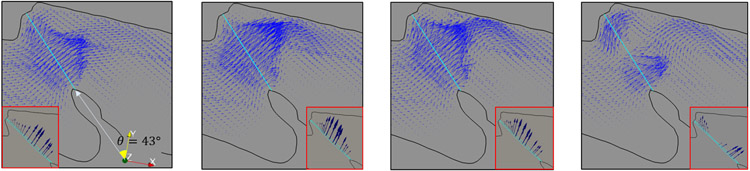
Time sequence of flow for Sample 3. Left to right: *t* = 0.56, 0.6, 0.64, and 0.66 cs. Inset figures show the corresponding glottal shape along with the velocity vectors through the glottis.

Figure 9 shows several time snapshots from *t* = 0.56 to 0.66 cs, representing vocal fold opening, fully open, closing, and fully closed, respectively. An important observation is that since the flow tends to follow the generally reversed *Z*-shaped geometry of the larynx, the majority of the flow is directed toward the anterior side after exit the glottis. This skewed direction forms the major cause for the flow asymmetry in supraglottal region. Fortunately, the vocal fold has an inclined orientation with respect to the larynx and remains approximately perpendicular to the flow direction. This alignment facilitates the interaction between the vocal fold and the flow and is beneficial to the flow-induced vibration. The inclination angle *θ* is illustrated in Figure 9, and Table 3 lists the value of *θ*, which is between 20 and 45 degrees for all five samples.

Due to the flow skewness, a large circulation persists on the posterior side of the supraglottal region throughout the vibration cycle, and this major vortex is strengthened as the flow speed increases following the vocal fold opening. On the other hand, a much smaller vortex may develop at the anterior side, e.g., at *t* = 0.62 cs when the vocal fold is closing. This vortex is due to closure of the anterior side of the glottis while the posterior side remains open (the posterior side of the vocal fold has minimal displacement and does not close completely), allowing the flow to continue going through. Such a vortex pair is repetitively observed at the start of vocal fold closure during each cycle.

**Figure 10.**
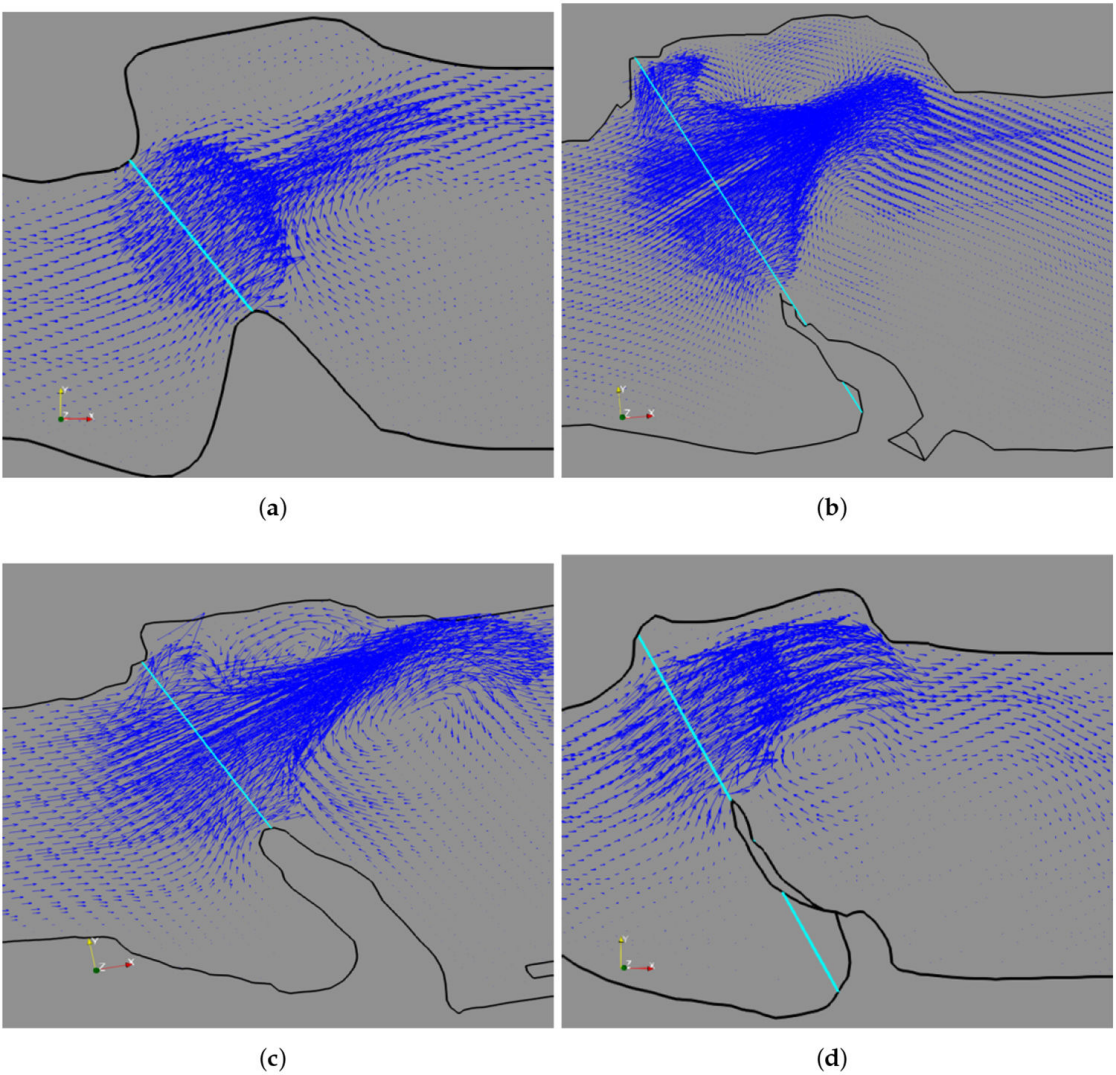
Flow pattern in the supraglottal region and the anterior, posterior vortices in Samples 1, 2, 4, and 5 in (**a–d**) respectively. The vocal fold is at an open state.

In addition to Sample 3, we also have a similar observation for the other four samples, which are shown in Figure 10. In this figure, we see the notable geometry differences among the samples, especially in the alignment of the subglottal and supraglottal channels. The anterior-end vortex in the supraglottal region is prominent in Samples 1, 2, and 4 due to a protruded space in the region. In all the five samples, the large posterior-end vortex is distinctly identified. This flow feature is caused by the overall orientation of the subglottal-supraglottal channels and inclination of the vocal fold. Such anterior-posterior flow asymmetry was also reported in a subject-specific computational analysis of the human larynx due to the similar geometric asymmetry [24], and it was believed that the flow asymmetry produced the anterior-posterior (longitudinal) asymmetry in the vocal fold vibration. In our study, we also observed longitudinal asymmetry in the vibration. However, there may be additional factors that have caused this behavior, for example, varying shapes of the vocal fold tissue structure in the transverse plane along the longitudinal direction, as well as differences in the cartilages that the vocal fold is attached to at the anterior and posterior ends. Further study may be needed to better understand the longitudinal differences in the vibration.

**Figure 11.**
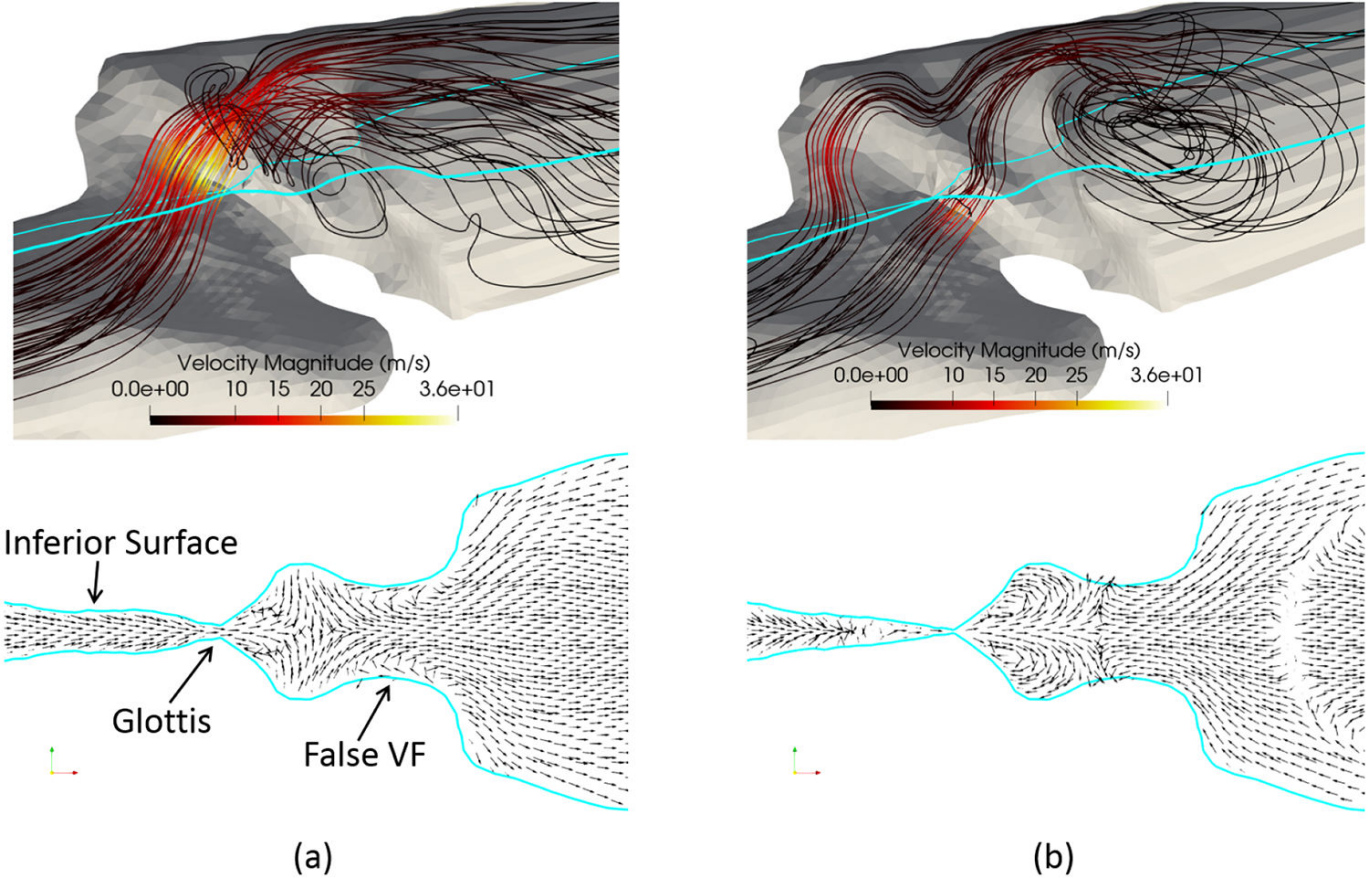
3D flow pattern in Sample 3 at (**a**) vocal fold opening (*t* = 0.6 cs) and (**b**) vocal fold closing (*t* = 0.66 cs). Bottom row: corresponding velocity field in the cut plane (blue lines) shown in the top row.

There have been extensive discussions on the supraglottal flow jet symmetry in the lateral direction. Previously, studies conducted both numerically and experimentally have reported varying asymmetrical glottal jet in the lateral direction when using simplified larynx models [9,39-42], and the phenomenon was explained using the bifurcation of the confined jet in a large expansion [39]. It was found that the presence of the false vocal fold helps reduce the expansion ratio of the geometry and maintain the symmetry of the flow [40,43]. The subject-specific 3D FSI study by Xue et al. [24] observed a fairly symmetrical jet flow in the lateral direction during most of the vibration cycle, which is consistent with the effect of the false vocal fold. In the current study, we report a symmetrical downstream flow pattern in the lateral direction, owing majorly to similar geometrical factors. In Figure 11, a 3D view of the flow is shown at *t* = 0.6 cs (open) and *t* = 0.66 cs (closed) stages, respectively. For each 3D view, a *YZ* cut plane is made, in which the velocity vectors downstream the vocal fold are shown. These pictures show the lateral symmetry of the flow domain and the presence of the false vocal fold helps maintain the flow symmetry in the lateral direction.

At the open condition (Figure 11A), we see that some of the flow is reversed in the ventricle region before the false vocal fold, which is due to the circulation of two lateral vortices in that region. Further downstream, a fairly uniform and symmetric outgoing flow is formed. At the closed condition (Figure 11B), we see in the cut plane that the two lateral vortices are still present in the ventricle region. Between the false vocal fold and immediately downstream, there is significant reversal flow due to the large posterior-end vortex. Despite the strong asymmetry in the anterior-posterior direction that was discussed early, the flow is able to retain symmetry in the lateral direction.

The 3D characteristic flow patterns observed here have significant similarity to those from human phonation, e.g., the anterior-posterior asymmetry and the lateral symmetry. This finding has important implications. Our current efforts are focused on the study of vocal fold vibration in the unilateral vocal fold paralysis (UVFP) as well as in the implanted configuration that aims to improve the implant design for individual subjects. The rabbit and its evoked phonation are being used in such efforts before similar studies can be done on human subjects. One important consideration in the UVFP and implant study is the restoration of symmetric vibration between the left and right sides of the vocal fold and the lateral symmetry in the flow. The present findings from the 3D analyses would help support the use of the rabbit as an intermediate step toward a human study.

## 4. Conclusions

A full 3D FSI simulation has been performed on the five subject-specific samples of the rabbit larynx, which were previously used for in vivo phonation experiments and 1D-flow based FSI study. These samples vary in size, geometry, and tissue properties, and are subjected to different inlet flow pressure during evoked phonation. Through the simulation, we obtained the detailed vibration characteristics and 3D flow pattern. The simulation-predicted vibration agrees with the experiment result for each individual subject. Furthermore, both the vocal fold displacement and intraglottal pressure agree with those from the 1D-flow model based simulation.

In the present rabbit phonation study, it was found that the gentle-slope inferior surface of the vocal fold plays a major role in the energy transfer between the fluid to the vocal fold tissue during vibration. For the flow field, we found that the flow in the supraglottal region is significantly skewed to the anterior side due to the asymmetrical geometry of the larynx and inclination of the vocal fold with respect to the axis of the larynx. However, the flow is fairly symmetric in the lateral direction thanks to the presence of the false vocal fold, which limits the expansion ratio of the geometry for the flow exiting the glottis. Such 3D flow patterns are generally consistent with those found previously in subject-specific modeling of healthy human phonation. Therefore, the use of rabbits may provide helpful insight into the study of human phonation.

## Supplementary Material

Supplemental Materials

## Figures and Tables

**Table 1.** Number of tetrahedral elements in the finite-element model of the vocal fold.

Sample	1	2	3	4	5
Elements	40,394	63,070	84,368	101,166	81,386

**Table 2.** Inlet pressure in the experiments and Young’s modulus for the vocal fold body, *E_b_*, and for the vocal fold cover, *E_c_*, which were previously determined for each individual samples (Data from [27]).

Sample	1	2	3	4	5
Pressure (kPa)	1.05	0.78	0.72	1.0	0.98
*E_b_* (kPa)	60	80	80	90	90
*E_c_* (kPa)	12	8	8	9	9

**Table 3.** VF inclination angle *θ* from the transverse axis (*Y*-axis).

Sample	1	2	3	4	5
*θ* (degrees)	41	24	43	20	29

**Figure A1.**
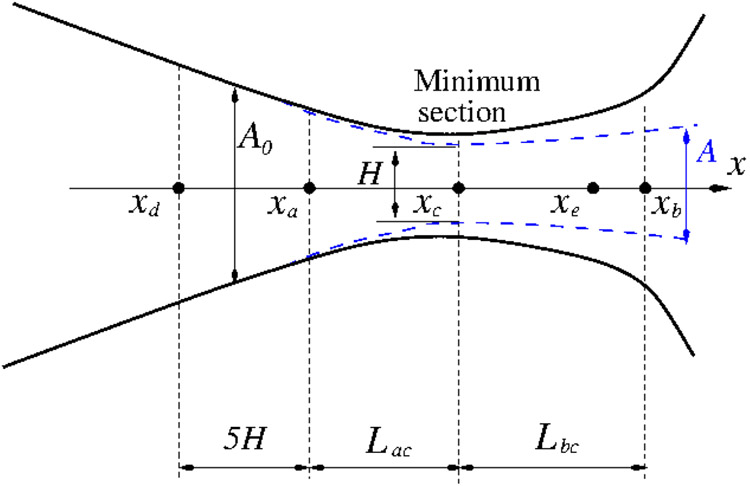
Schematic for the 1D pulsatile flow model, where the characteristics of vocal fold are described by a simple 2D converging-diverging channel. Here *x_c_* is the narrowest section, *x_a_* is an inferior location, *x_b_* is the glottal exit, *x_e_* is an intermediate location in the diverging section, and *x_d_* is a subglottal location.

**Figure A2.**
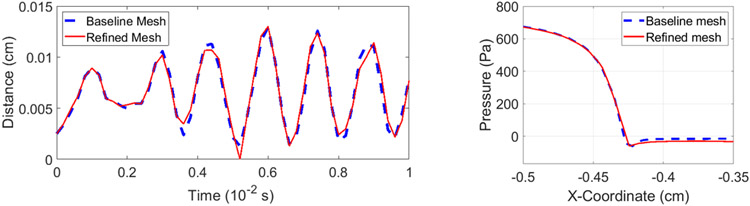
**Left:** vocal fold displacement; **right:** intraglottal pressure plot at *t* = 0.6 cs for the baseline and refined meshes (Sample 3).

## Data Availability

The presented data in the current study is available on request from the authors. Final datasets will be publicly available when complete, per the resource sharing plan described in NIDCD award #5R01DC016236.

## Appendix

### Appendix A. Additional Detail for the 1D-Flow Based FSI Model

As described in Section 2.3, the 1D pusatile flow model is given by Equation (1). Further details of the entrance effect to correct the area, and the pressure loss term, *τ*, are summarized here. To correct the cross-sectional area due to the contraction effect at the glottal entrance, we define a correction coefficient, *α*, so that *α*(*x*) = *A*(*x*)/*A*_0_(*x*), where *A*_0_(*x*) is the actual cross-sectional area as shown in Figure A1.

In the 1D model, *α*(*x*) is assumed to be a parabolic function with *α* = 1 at *x_c_* and *dα/dx* = 0 at *x_b_*. Thus, only *α*(*x_b_*) needs to be determined. For the pressure loss, *τ*(*x_e_*) and *τ*(*x_b_*) are to be determined, and *τ*(*x*) follows an assumed 3rd-order polynomial function between *x_a_* and *x_b_*.

The nondimensional variables describing the flow are the Reynolds number *Re*, the divergence angle *r_b_*, the length of the divergence section *l_bc_*, the inferior section angle *r_a_*, the length of the convergence section *l_ac_*, and the subglottal section angle *r_d_*, which are defined as

(A1)
$$r_{b}\;=\;A(x_{b})∕A(x_{c}),\;l_{bc}=L_{bc}∕H,\;\;r_{a}=A(x_{a})∕A(x_{c}),\;\;l_{ac}=L_{ac}∕H,\;\;r_{d}=A(x_{d})∕A(x_{a}),\;Re\;=\;\rho \;u_{c}\;H∕\mu$$

In the machine learning model, the nondimensionalized variables, *α*(*x_b_*), *τ_e_*, and *τ_b_* are expressed as functions of the above input variables,

(A2)
$$f=f(r_{b},\;l_{bc},\;r_{a},\;l_{ac},\;r_{d},\;Re^{1∕3},\;r_{b}^{2},\;r_{b}l_{bc},\cdots ,\;Re^{2∕3},\cdots )$$

The explicit functions derived from machine learning are given in Li et al. [34].

Tetrahedral elements consisting of 10 nodes help in capturing the intricate geometry for finite element model [44,45]. The exterior surfaces in the finite element model of the larynx are fixed, whereas the lumen surface is free. As described in the previous work by Chang et al. [27], the mesh sensitivity challenge from the thin VF cover layer was addressed by determining two layers for the VF cover. The use of a similar structure type was also reported by Alipour et al. [7] and Zhang et al. [46]. Note that the material properties adopted for VF body and cover in the previous 1D FSI study [27] were identified based on the finite element model used. This restricts the model modifications for the current 3D FSI analysis. This process can be further improved by meshing individual segmented part but the integration of such a model in the current 3D FSI analysis and computational cost incurred need to be explored.

### Appendix B. Mesh Independence Study

A mesh-independence study for Sample 3 is shown in the Figure A2 with the baseline mesh for the flow formed by a non-uniform Cartesian grid with 211 × 129 × 133 points and the refined mesh with 371 × 154 × 144 points in the *X*, *Y*, and *Z* coordinates. The displacement of a point at the mid-glottis is used to compare between the two mesh settings. In addition, the pressure along the streamline in Figure 8a is compared between the two FSI simulations at *t* = 0.6 cs when the VF is fully opened. In both the displacement and the intraglottal pressure comparisons, the baseline-mesh result agrees well with the result from the refined mesh. The mean error in the pressure value between the two meshes is 4%.
